# 1.3 kV Vertical GaN-Based Trench MOSFETs on 4-Inch Free Standing GaN Wafer

**DOI:** 10.1186/s11671-022-03653-z

**Published:** 2022-01-15

**Authors:** Wei He, Jian Li, Zeliang Liao, Feng Lin, Junye Wu, Bing Wang, Maojun Wang, Nan Liu, Hsien-Chin Chiu, Hao-Chung Kuo, Xinnan Lin, Jingbo Li, Xinke Liu

**Affiliations:** 1 College of Materials Science and Engineering, College of Electronics and Information Engineering, College of Physics and Optoelectronic Engineering, Institute of Microelectronics (IME), Shenzhen University, Shenzhen, 518060 China; 2 Institute of Microelectronics, Peking University, Beijing, 100871 China; 3 Shaanxi Reactor Microelectronics Co., Ltd., Xi’an, 710075 China; 4 Department of Electronic Engineering, Chang Gung University, Taoyuan, 333 Taiwan; 5 Department of Photonic and Institute of Electro-Optical Engineering, National Chiao Tung University, Hsinchu, 300 Taiwan; 6 The Shenzhen Key Lab of Advanced Electron Device and Integration, ECE, Peking University Shenzhen Graduate School, Shenzhen, 518055 China; 7 Institute of Semiconductors, South China Normal University, Guangzhou, 510631 Guangdong China

**Keywords:** Free standing gallium nitride (GaN), Trench MOSFET, GaN MOSFET, Breakdown, TCAD

## Abstract

In this work, a vertical gallium nitride (GaN)-based trench MOSFET on 4-inch free-standing GaN substrate is presented with threshold voltage of 3.15 V, specific on-resistance of 1.93 mΩ·cm^2^, breakdown voltage of 1306 V, and figure of merit of 0.88 GW/cm^2^. High-quality and stable MOS interface is obtained through two-step process, including simple acid cleaning and a following (NH_4_)_2_S passivation. Based on the calibration with experiment, the simulation results of physical model are consistent well with the experiment data in transfer, output, and breakdown characteristic curves, which demonstrate the validity of the simulation data obtained by Silvaco technology computer aided design (Silvaco TCAD). The mechanisms of on-state and breakdown are thoroughly studied using Silvaco TCAD physical model. The device parameters, including n^−^-GaN drift layer, p-GaN channel layer and gate dielectric layer, are systematically designed for optimization. This comprehensive analysis and optimization on the vertical GaN-based trench MOSFETs provide significant guide for vertical GaN-based high power applications.

## Introduction

Wide-bandgap GaN-based power devices have been regarded as the great potential candidates for the next generation efficient power electronics and compact power systems, owing to the superior material properties such as high electron mobility, large breakdown field strength and high thermal stability [1–5]. Compared with high electron mobility transistors (HEMTs) [6–11] and current aperture vertical electron transistors (CAVETs) [12–15], GaN-based trench metal oxide semiconductor field effect transistors (MOSFETs) [16–18] are more competitive to realize intrinsically normally-off operation with higher current density, lower specific on-resistance (*R*_on,sp_) and lower current collapse. Moreover, GaN-based trench MOSFETs possess relatively simple manufacturing process and do not need the regrowth of AlGaN/GaN layers [19, 20].

The development of lateral GaN-based MOSFETs has approximately come to saturation, due to the breakdown voltage (*V*_BR_) limited by the length of lateral drift region. Although the growth of length can increase *V*_BR_, the size of device enlarges, leading to reduction of the effective current density per unit chip area. In contrast, vertical GaN-based devices have been fully advanced. Under the same required *V*_BR_ and amperage rating, smaller size and less cost can be realized on vertical GaN-based MOSFETs when make a contrast with lateral GaN MOSFETs [21]. In comparison with Si, Sapphire, SiC and Diamond substrate, the MOSFETs on free-standing GaN substrate can greatly reduce the probability of the high-density trap states and non-linearity contributed by lattice mismatch while operating at high power [22].

More studies have made great progress in *V*_BR_, *R*_on,sp_ and device reliability for GaN vertical MOSFETs in recent years. Floating P-body had been introduced in the N^−^-GaN drift region to form “P-body/N-drift” junction via TCAD simulation for the improvement of *V*_BR_ of the enhancement-mode vertical GaN MOSFET [23]. Vertical GaN interlayer-based trench MOSFET (OG-FET) on a large-area in-situ oxide performed threshold voltage (*V*_th_) of 2.5 V, *R*_on,sp_ of 0.98 mΩ·cm^2^ and *V*_BR_ of 700 V with regrown 10-nm unintentional-doped-GaN interlayer as the channel and 50-nm in-situ Al_2_O_3_ as the gate dielectric [24]. Vertical GaN trench-MOSFETs with MBE regrown UID-GaN channel were investigated, which avoided the need to reactivate the buried body p-GaN and promised the same benefit on channel mobility compared to the MOCVD regrowth [25]. The device characteristics had been improved for vertical GaN trench MOSFETs by using Silvaco ATLAS 2-D simulation in order to get the best trade-off between *V*_BR_ and *R*_on,sp_ [26].

In this work, we present vertical GaN-based trench gate MOSFETs (GaN TG-MOSFETs) on 4-inch free-standing GaN substrate exhibiting normally off operation for high power applications. We use Silvaco TCAD to simulate the structure and performance of GaN TG-MOSFETs based on semiconductor physics and advanced process. The simulation results obtained by Silvaco ATLAS simulation are consistent well with experiment data on the characteristic curves of transfer, output, and breakdown voltage, respectively. The device parameters are researched comprehensively by using TCAD for providing guide in actual fabrication and optimization. The design of the parameters includes the thickness of n^−^-GaN drift body layer (*L*_drift_), n^−^-GaN drift trench region (*L*_trench_), p-GaN channel layer (*L*_channel_) and gate dielectric layer (*L*_dielectric_). The doping density of p-GaN channel layer (*N*_a_) and n^−^-GaN Drift layer (*N*_d_) are included.

## Experiment and Simulation Approach

**Fig. 1:**
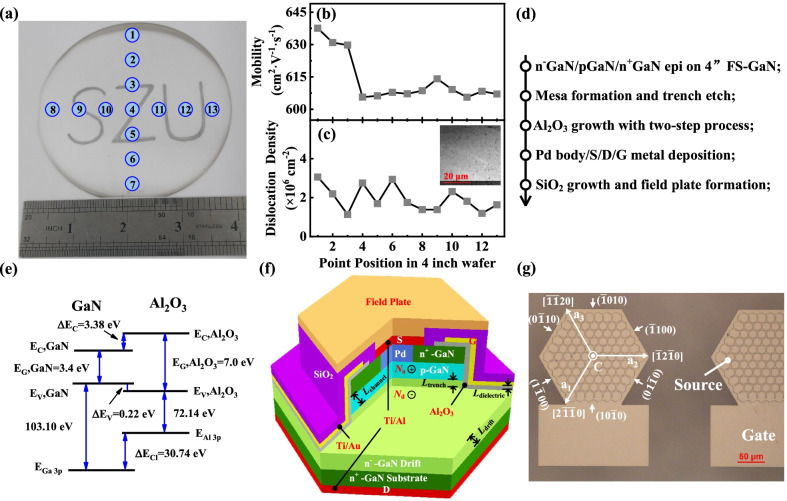
**a** Photograph of 4-inch freestanding GaN wafer, where the letters SZU can be clearly seen. **b** The mobility of GaN wafer. **c** The dislocation density and CL image of the epitaxial layer. **d** The fabrication process. **e** The schematic of energy band lined-up at the Al_2_O_3_/GaN heterointerface. **f** 3D drawing structure and **g** micrograph of the fabricated GaN TG-MOSFET

High-quality, large-size and less-expensive GaN substrates are crucial for the progress of vertical GaN power devices. More techniques were proposed to optimize the growth of bulk GaN crystals, such as halide vapor phase epitaxy (HVPE), high nitrogen pressure solution (HNPS), basic and acidic ammonothermal, Na-flux method and near atmospheric pressure solution growth [27, 28]. HVPE is the main method for mass fabrication of GaN crystals, due to its high grow rate, high purity, high process repeatability and easy doping. The transparent 4-inch freestanding GaN wafer grown by HVPE with 13 points position for test is shown in Fig. 1a. We utilized a 420-μm-thick free-standing n^+^-GaN substrate in the device fabrication with the average mobility of 614 cm^2^·V^−1^·s^−1^ and the average dislocation density of 1.94 × 10^6^ cm^−2^ at the top surface, as determined by contactless Hall measurement and cathodoluminescence (CL). The test result and CL image of the epitaxial layer are presented in Fig. 1b, c, respectively.

The fabrication process of the GaN TG-MOSFETs discussed in this work is shown in Fig. 1d. The epitaxial growth began with 12-*µ*m lightly doped 8.0 × 10^15^ cm^−3^ n^−^-GaN as the drift region. A 1.0-*µ*m heavily doped p-GaN with a doping density of 1.0 × 10^18^ cm^−3^ was deposited as the channel region. Thereafter, a 0.2-*µ*m-thick heavily doped n^+^-GaN with a doping density of 3 × 10^18^ cm^−3^ was grown as the source contact layer. The device fabrication process started with the formation of 0.2-μm-deep vertical trench and 1.7-μm-deep vertical mesa for p-body and gate contacts by using Cl_2_-based gases in reactive ion etching (RIE) at 15 W power, respectively. A 16-nm-thick Al_2_O_3_ film was deposited by atomic layer deposition (ALD) as gate dielectric. High-quality and stable MOS interface with low-density trap states is essential for GaN TG-MOSFETs. A two-step process, including simple acid cleaning and a following (NH_4_)_2_S passivation, was required to drastically reduce the interface states and border traps [29]. The source and drain electrodes with Ti/Al were annealed at 550 °C for 5 min in N_2_ ambient for ohmic contacts. The gate and p-body electrodes were composed of Ti/Au and Palladium, respectively. A 400-nm-thick SiO_2_ film was deposited by plasma enhanced chemical vapor deposition (PECVD) as the passivated isolation mesa. Finally, field plate termination was employed to impair the peak electric field crowded at the edge of PN junction around the isolation mesa. The Al-based field plate was connected to the source electrode.

The schematic of energy band lined-up at the Al_2_O_3_/GaN heterointerface in Fig. 1e. The forbidden band of GaN was exactly contained in that of Al_2_O_3_, where the deviations of the conduction band and valence band were 3.38 eV and 0.22 eV, respectively. It revealed that Al_2_O_3_ could maintain excellent insulation with GaN for electrons, which greatly reduced gate leakage current and improved device performance. The 3D drawing structure and parameters needed for optimization are shown in Fig. 1f. Figure 1g shows the micrograph of the device. The hexagonal crystal structure contained the outward vertical C axis, three horizontal axes a_1_, a_2_ and a_3_, and crystal planes in various directions.

The physical simulation models concerned for simulation were the parallel electric field-dependent mobility model, concentration-dependent mobility model, low field mobility model, Shockley–Read–Hall recombination model, Auger recombination model, impact ionization model, energy bandgap narrowing model and trap model [26, 29–31]. The main physical models and parameter values for simulation are shown in Table 1.

**Table 1:** The main model and parameter values for TCAD simulation

Model	Parameter	Value	Unit
Incomplete ionization	*g*_*D*_	2	–
	*E*_*D,0*_	0.017	eV
	*θ*_*n*_	3.4 × 10^–9^	eV·cm
	*g*_*A*_	2	–
	*E*_*A,0*_	0.16	eV
	*θ*_*p*_	3.14 × 10^–8^	eV·cm
Low field mobility	*µ*_*1n*_	250	cm^2^/V·s
	*µ*_*2n*_	1150	cm^2^/V·s
	*N*_*refn*_	2 × 10^17^	cm^−3^
	*ρ*_*n*_	1.0	–
	*µ*_*1p*_	10	cm^2^/V·s
	*µ*_*2p*_	170	cm^2^/V·s
	*N*_*refp*_	3 × 10^17^	cm^−3^
	*ρ*_*p*_	2.0	–
Impact ionization	*AN*	2.1 × 10^9^	cm^−1^
	*BN*	3.4 × 10^7^	cm^−1^
	*AP*	5.4 × 10^6^	V/cm
	*BP*	1.8 × 10^7^	V/cm
	*BETAN*	1.0	–
	*BETAP*	1.0	–

For GaN simulation, Si donors and Mg acceptors were not completely ionized at room temperature since their high activation energies, especially for Mg-doped p-type GaN [32–34]. Thus, according to Fermi–Dirac distribution, the incomplete ionization model was incorporated in the simulation for accurately reproducing the breakdown voltage. The ionized donors and acceptors impurity concentrations were given as follows

1 $$N_{D}^{ + } = \frac{{N_{D} }}{{1 + g_{D} \cdot \exp (\frac{{\varepsilon_{Fn} + E_{D,0} - \theta_{n} \cdot \sqrt[3]{{N_{D}^{{}} }} - E_{C} }}{KT})}}$$

2 $$N_{A}^{ - } = \frac{{N_{A} }}{{1 + g_{A} \cdot \exp (\frac{{E_{V} + E_{A,0} - \theta_{p} \cdot \sqrt[3]{{N_{A}^{{}} }} - \varepsilon_{Fp} }}{KT})}}$$

Here, *g*_*D*_ and *g*_*A*_ are the appropriate degeneracy factors for conduction and valence bands. *E*_*D,0*_ and *E*_*A,0*_ are the donor and acceptor ionization energy at very low doping levels. *θ*_*n*_ and *θ*_*p*_ are constants accounting for geometrical factors as well as for the properties of the material. Low field mobility model is the result of fitting Caughey Thomas like model to Monte Carlo data [26, 32]. It can be defined as

3 $$\begin{aligned} \mu_{(n/p)} (T,N) & = \mu_{1(n/p)} \cdot (T/300)^{{\beta_{1(n/p)} }} + \\ & \frac{{(\mu_{2(n/p)} - \mu_{1(n/p)} ) \cdot (T/300)^{{\beta_{2(n/p)} }} }}{{1 + [\frac{N}{{N_{ref(n/p)} \cdot (T/300)^{{^{{\beta_{3(n/p)} }} }} }}]^{{\rho_{(n/p)} \cdot (T/300)^{{^{{^{{\beta_{4(n/p)} }} }} }} }} }} \\ \end{aligned}$$

where *µ*_1_, *µ*_2_ are the minimum and maximum mobility, *ρ*, *β*_1_, *β*_2_, *β*_3_, *β*_4_, are all temperature dependent fitting parameters, *N*_*ref*_ is the reference doping level and *N* is the donor concentration.

The Poisson’s equations and Current continuity equation were essential for the analysis of simulation [35]. As in semiconductor PN junction, avalanche breakdown occurred when the impact ionization integral reached unity

4 $$I_{n} = \int {\alpha_{n} \exp \left(\int_{{}}^{w} {\alpha_{p} - \alpha_{n} {\text{d}}v} \right)} {\text{d}}w = 1$$

where *I*_n_ is the impact ionization integral of electrons. The utilized ionization rate model of electrons and holes are variation of the classical Chynoweth model [36], which based upon the following expressions,

5 $$\alpha_{n} = AN \cdot \exp \left[ { - \left( \frac{BN}{E} \right)^{BETAN} } \right]$$

6 $$\alpha_{p} = AP \cdot \exp \left[ { - \left( \frac{BP}{E} \right)^{BETAP} } \right]$$

Here, *E* is the electric field in the direction of current flow at the p-GaN channel layer in the structure. Various group has reported impact ionization coefficients to accurately predict the breakdown of GaN power devices in recent years [37–41]. The coefficients *AN*, *AP*, *BN*, *BP*, *BETAN* and *BETAP* of the impact ionization model in this work were determined by referring to the experiments above.

In this study, the work was mainly carried out by TCAD. The data obtained by simulation had been calibrated with the result of experiment on GaN TG-MOSFET shown in the third part. The comprehensive analysis and optimization design on *L*_drift_, *L*_trench_, *L*_dielectric_, *L*_channel_, *N*_a_ and *N*_d_ of devices were demonstrated in the fourth part, respectively.

## Results and Discussion

**Fig. 2:**
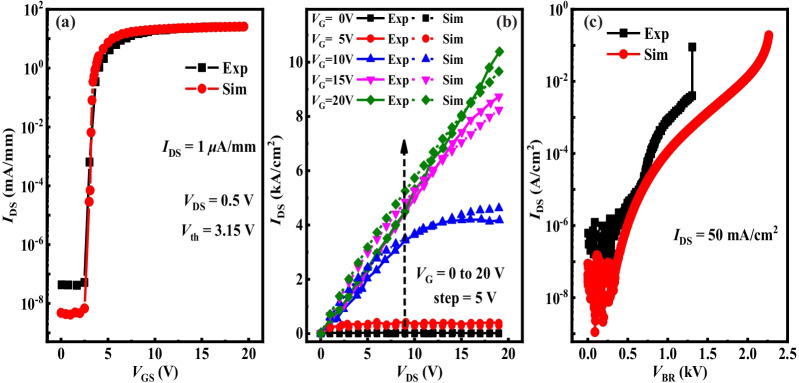
**a** Transfer *I*–*V* characteristics (*I*_D_–*V*_G_) at *V*_DS_ = 0.5 V. **b** Output *I*–*V* characteristics (*I*_D_–*V*_D_) at *V*_GS_ = 0 V, 5 V, 10 V, 15 V and 20 V, respectively. **c** Off-state *I*–*V* characteristics measured at *V*_G_ = 0 V for fabricated GaN TG-MOSFET

The initial device parameters of simulation model were set as follows: *N*_d_ = 8.0 × 10^15^ cm^−3^, *N*_a_ = 1.0 × 10^18^ cm^−3^*, L*_drift_ = 12 μm*, L*_trench_ = 0.5 μm*, L*_channel_ = 1.0 μm, and *L*_dielectric_ = 16 nm. The interface state could capture the free electrons in the channel and formed the negative interface charge, leading to the decrease of the number of free electrons and the increase of *R*_on,sp_. A low density of interface state was beneficial to reduce *R*_on,sp_ and switch loss. The interface state in the simulation was defined as 10^11^ cm^−2^·eV^−1^ by referring to the previous work [25]. The characteristic curves of transfer, output, and breakdown of GaN TG-MOSFET via experiment (Exp) and simulation (Sim) are shown in Fig. 2, respectively. This simulation results were consistent well with the data of experiment, which could verify the validity of the results obtained by simulation and calibrate the simulation model.

**Fig. 3:**
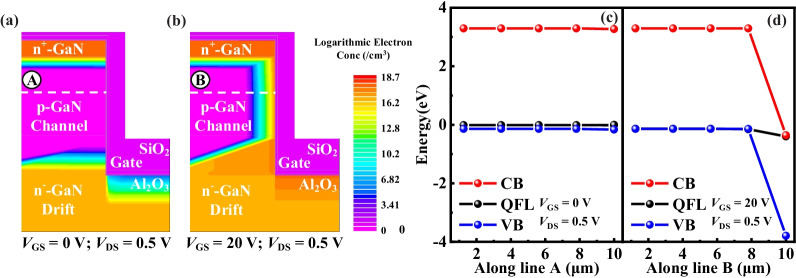
Electron concentration distributed at **a** *V*_GS_ = 0 V, *V*_DS_ = 0.5 V (off-state) and **b** *V*_GS_ = 20 V, *V*_DS_ = 0.5 V (on-state). The energy band distributed during **c** off-state and **d** on-state

Figure 2a shows the *I*_D_–*V*_G_ characteristics at *V*_DS_ = 0.5 V. Several extraction methods were used to determine the value of *V*_th_ from the measured *I*_D_–*V*_G_ characteristics [42]. Normally-off operation with *V*_th_ (defined at *I*_DS_ = 1 μA/mm) of 3.15 V was observed. Figure 3a shows the current could not be conducted between the source and drain, since the channel was not yet formed a conduction path. In Fig. 3b, the inversion layer of electron was effectively generated in the channel only when *V*_GS_ > *V*_th_, hence generating the drain-to-source current. The distributions of energy band along line A and line B during off-state and on-state are shown in Fig. 3c and Fig. 3d, respectively. From off-state to on-state, the energy of conduction band (CB) obviously reduced until closing to the Quasi-Fermi level (QFL). Therefore, electrons could easily jump to CB and generate conduction current. Figure 2b exhibits the output *I*–*V* characteristics at *V*_GS_ = 0 V, 5 V, 10 V, 15 V and 20 V, respectively. The *R*_on,sp_ estimated from the linear region was 1.93 mΩ·cm^2^ at *V*_DS_ = 0.5 V and *V*_GS_ = 20 V.

Figure 2c demonstrates the off-state *I*–*V* characteristics measured at *V*_GS_ = 0 V. This work achieved the hard breakdown *V*_BR_ of 1306 V when *I*_DS_ > 50 mA/cm^2^ from experiment, while the *V*_BR_ of simulation reached 2278 V. The insufficient activation of the Mg dopant existing in the p-GaN was considered as the reason for making the discrepancy in *V*_BR_ between experiment and simulation. Finally, the corresponding figure of merit (FOM) obtained were 0.88 GW/cm^2^ and 1.68 GW/cm^2^ by experiment and simulation, respectively.

**Fig. 4:**
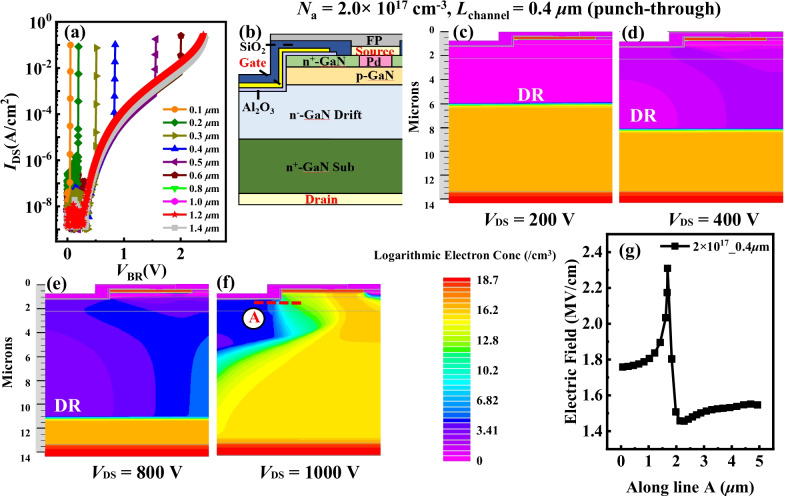
**a** Breakdown curves under *N*_a_ = 2.0 × 10^17^ cm^−3^ and various *L*_channel_. **b** Schematic of GaN TG-MOSFET. **c**–**f** Depletion region at different reverse bias under *N*_a_ = 2.0 × 10^17 ^cm^−3^ and *L*_channel_ = 0.4 μm, including *V*_DS_ = 200 V, 400 V, 800 V and 1000 V. **g** Electric Field distributed along line A at *V*_DS_ = 1000 V

**Fig. 5:**
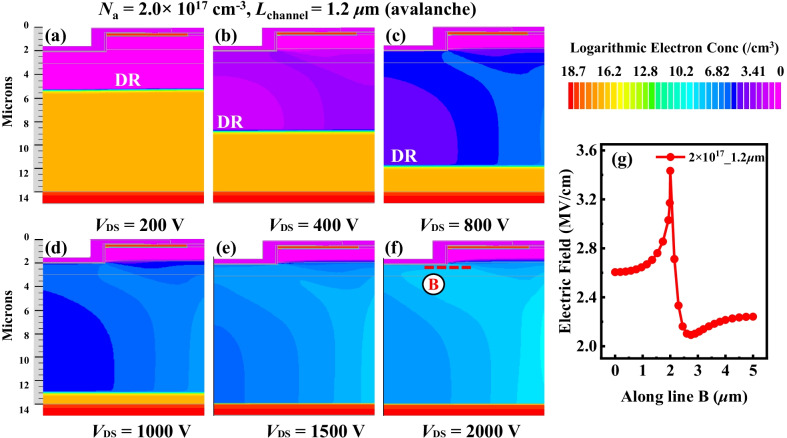
**a**–**f** Depletion region at different reverse bias under *N*_a_ = 2.0 × 10^17^ cm^−3^ and *L*_channel_ = 1.2 μm, including *V*_DS_ = 200 V, 400 V, 800 V, 1000 V, 1500 V and 2000 V. **g** Electric Field distributed along line B at *V*_DS_ = 2000 V

Two breakdown mechanisms, namely punch-through breakdown and avalanche breakdown, existed in the device, which were dominated by the product of *L*_channel_ and *N*_a_ (*L*_channel_ · *N*_a_) of p-GaN. Taking the *N*_a_ = 2.0 × 10^17^ cm^−3^ and various *L*_channel_ for example by simulation as shown in Fig. 4a. Punch-through breakdown would occur when *L*_channel_ · *N*_a_ was lower than a certain value, such as *L*_channel_ = 0.6 μm and *N*_a_ = 2.0 × 10^17^ cm^−3^ in Fig. 4a. However, it would be changed to avalanche breakdown when *L*_channel_ · *N*_a_ was high enough, such as *L*_channel_ = 0.8 μm and *N*_a_ = 2.0 × 10^17^ cm^−3^. Moreover, the breakdown mechansims were studied in detail from the expansion of depletion region (DR), distributions of electric field and impact gen rate (IGR). Figure 4b shows the schematic of the device. Figures 4c–f and 5a–f show the expansion of DR of device with *L*_channel_ = 0.4 μm and *L*_channel_ = 1.2 μm, respectively. As drain voltage enlarged, the DR extended continually and was oriented toward the drain, which offered strong current blocking capability and suppressed the permature breakdown.

**Fig. 6:**
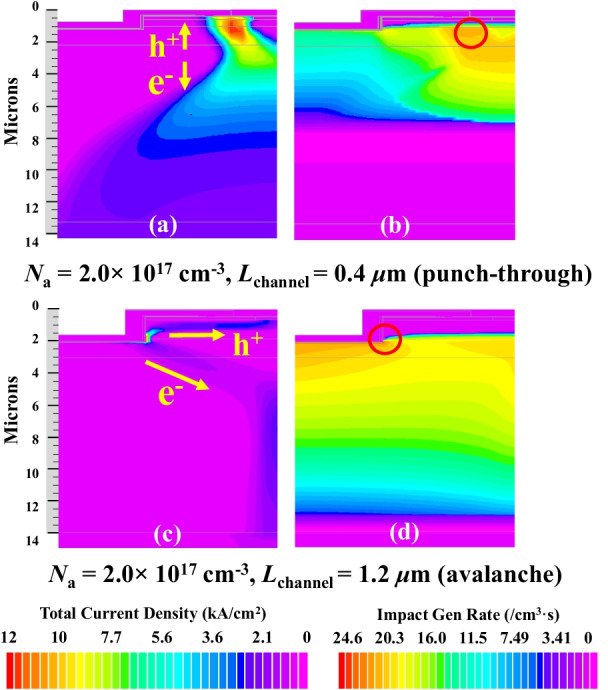
**a**, **b** were the distributions of Total Current Density and Impact Gen Rate under *N*_a_ = 2.0 × 10^17^ cm^−3^ and *L*_channel_ = 0.4 μm, respectively. **c**, **d** were the distributions of Total Current Density and Impact Gen Rate under *N*_a_ = 2.0 × 10^17^ cm^−3^ and *L*_channel_ = 1.2 μm, respectively

Punch-through breakdown occured in the device with *N*_a_ = 2.0 × 10^17^ cm^−3^ and *L*_channel_ = 0.4 μm when *V*_DS_ > 1000 V for *L*_channel_ · *N*_a_ was low. The EF along line A is shown in Fig. 4g. The peak EF was ~ 2.3 MV/cm and did not reach the critical electric field strength value of GaN. A high potential barrier prevents current flow. The barrier between source and drain significantly reduced for the expansion of DR when *V*_DS_ increased. Since the current was an exponential function of barrier height, the current increased rapidly once the punch-through condition was satisfied. The number of electrons injected from source region into channel had greatly increased through EF. Large current flowed from drain directly to source as shown in Fig. 6a. In Fig. 6b, the peak IGR was located at the reverse biased PN junction between p-GaN and n^−^-GaN drift region as shown in the red circle.

Avalanche breakdown happened in the device with *N*_a_ = 2.0 × 10^17^ cm^−3^ and *L*_channel_ = 1.2 μm when *V*_DS_ > 2000 V for the *L*_channel_ · *N*_a_ was high. The EF along line B is shown in Fig. 5g. The peak EF was ~ 3.45 MV/cm and closed to the critical electric field of 3.3 MV/cm of GaN. The energy of electrons and holes was enhanced by EF when they pass through the space charge region. Since they collided with electrons of atoms in DR, large numbers of new electron–hole pairs were generated, causing the avalanche effect. Large electron and hole current were produced as shown in Fig. 6c. The electron current mainly flowed to drain, while hole current flowed to the source along p-GaN region. In the red circle of Fig. 6d, the peak IGR was located at the gate corner. It implied that the breakdown characteristic was similar to that of PN junction diode as long as punch-through did not occur. The simulation showed that device could achieve avalanche and avoid punch-through breakdown with enough value of *L*_channel_·*N*_a_. The effect of various *N*_a_ on the breakdown mechanism was similar to that of *L*_channel_.

## Analysis and Performance Evaluation

This simulation focused on studying the effects of various device parameters and obtaining the scheme of optimization design. Firstly, the thickness and doping density of n^−^-GaN drift layer were researched with different initial values. Then, we analysed the thickness and doping density of p-GaN channel layer based on the optimal parameters of n^−^-GaN drift layer. Finally, the impact of the thickness of gate dielectric was thoroughly studied. All the changes of the above parameters were discussed within a reasonable range. The power figure of merit FOM = *V*_BR_^2^/*R*_on,sp_ and *V*_th_ could be used as a criterion for optimization.

### Analysis the Influence of n^−^-GaN Drift Layer

**Fig. 7:**
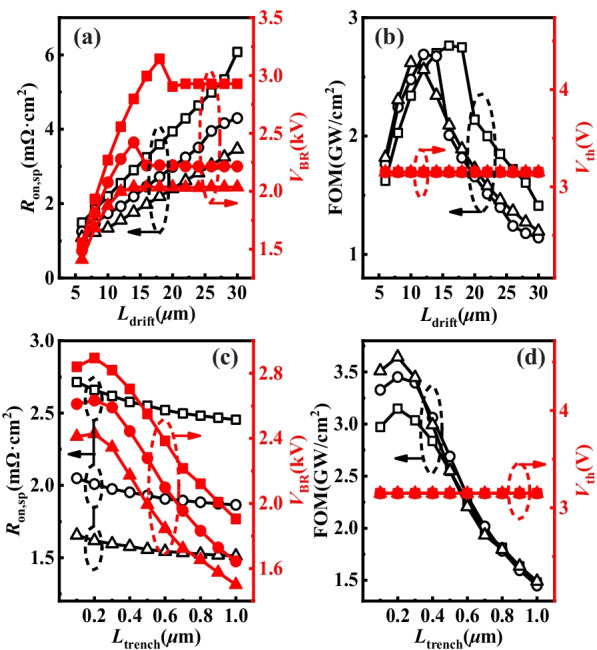
**a** Simulated *R*_on,sp_ and *V*_BR_ of the device versus *L*_drift_ and *N*_d_. **b** *V*_th_ and FOM as a function of *L*_drift_ and *N*_d_. **c** Simulated *R*_on,sp_ and *V*_BR_ of the device versus *L*_trench_ and *N*_d_. **d** *V*_th_ and FOM as a function of *L*_trench_ and *N*_d_. In (**a**)–(**d**), the square, circle and triangle curves were simulated under *N*_d_ = 6.0 × 10^15^ cm^−3^, *N*_d_ = 8.0 × 10^15^ cm^−3^ and *N*_d_ = 1.0 × 10^16^ cm^−3^, respectively

As shown in Fig. 7a, *V*_BR_ increased and saturated at a certain value as *L*_drift_ increased with different initial conditions. The phenomenon that DR extended and saturated with the growth of *L*_drift_ caused the *V*_BR_ increased and saturated. Conversely, *V*_BR_ decreased with the growth of *N*_d_. The situation was equivalent to the effect of the doping concentration in the low-doped side of the PN single junction diode on the *V*_BR_, which demonstrated that the value of *N*_d_ was inversely proportional to *V*_BR_. It implied that *V*_BR_ prematurely saturated at thin *L*_drift_ with high *N*_d._ Low *N*_d_ had larger DR than high *N*_d_ due to the IGR of electron was low. Large DR could withstand high voltage and prevent electron absorbing electric field energy to reach breakdown. The change of *R*_on,sp_ was mainly caused by the variation of *R*_drift_ (*R*_on,sp_ of n^−^-GaN drift layer). The *R*_on,sp_ decreased with the increase of *N*_d_ and decrease of *L*_drift_, respectively. *R*_on,sp_ decreased from 2.55 mΩ·cm^2^ to 1.56 mΩ·cm^2^ and *V*_BR_ reduced from 2558 to 1997 V as *N*_d_ increased from 8.0 × 10^15^ cm^−3^ to 1.0 × 10^16^ cm^−3^ under *L*_drift_ = 12 μm. In Fig. 7b, the obtained peak FOM were 2.76 GW/cm^2^, 2.69 GW/cm^2^ and 2.62 GW/cm^2^ under *L*_drift_ = 16 μm, *L*_drift_ = 12 μm, and *L*_drift_ = 10 μm with the growth of *N*_d_, respectively. It demonstrated that the optimal FOM could be obtained under the low *N*_d_ and thick *L*_drift_. In contrast, the change of *N*_d_ and *L*_drift_ had no effect on *V*_th_, and it remained at 3.15 V. The impact on *R*_on,sp_ and *V*_BR_ were obvious by the change of *N*_d_ and *L*_drift_, while hardly influenced *V*_th_.

With the increase of *L*_trench_ from 0.1 to 1.0 µm in Fig. 7c, d, *R*_on,sp_ continuously decreased. In contrast, *V*_BR_ and FOM first increased and then decreased as the advance of *L*_trench_, finally reaching peak value at *L*_trench_ = 0.2 µm. The obtained peak FOM were 3.15 GW/cm^2^, 3.45 GW/cm^2^ and 3.64 GW/cm^2^ under *N*_d_ = 6.0 × 10^15^ cm^−3^, *N*_d_ = 8.0 × 10^15 ^cm^−3^, and *N*_d_ = 1.0 × 10^16^ cm^−3^, respectively. The impact of *L*_trench_ on FOM was apparent when it was thin. The variety of *N*_d_ and *L*_trench_ also made little difference on *V*_th_. *V*_th_ showed independence for the change of *N*_d_*, L*_drift_ and *L*_trench_, due to the p-type channel region.

### Analysis the Impact of p-GaN Channal and Dielectric

**Fig. 8:**
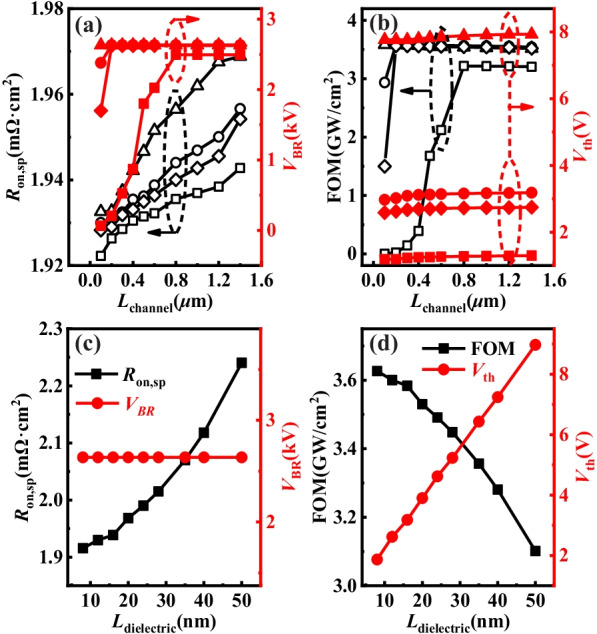
**a** Simulated *R*_on,sp_ and *V*_BR_ of the device versus *L*_channel_ and *N*_a_. **b** *V*_th_ and FOM as a function of *L*_channel_ and *N*_a_. In (**a**) and (**b**), the square, rhombus, circle and triangle curves were simulated under *N*_a_ = 2.0 × 10^17^ cm^−3^, *N*_a_ = 8.0 × 10^17^ cm^−3^, *N*_a_ = 1.0 × 10^18^ cm^−3^ and *N*_a_ = 3.0 × 10^18^ cm^−3^, respectively. **c** Simulated *R*_on,sp_ and *V*_BR_ of the device versus *L*_dielectric_. **d** *V*_th_ and FOM as a function of *L*_dielectric_

The effects of the *L*_channel_ and *N*_a_ of p-GaN channel layer were investigated based on *L*_drift_ = 12 μm and *L*_trench_ = 0.2 μm of n^−^-GaN drift layer. The change of *R*_on,sp_ was mainly caused by the variation of *R*_channel_ (*R*_on,sp_ of p-GaN channel layer). The curves of *R*_on,sp_ showed continuous rising trend with the enhancement of *L*_channel_ as shown in Fig. 8a. As *N*_a_ increased from 2.0 × 10^17^ cm^−3^ to 3.0 × 10^18^ cm^−3^ under *L*_channel_ = 1.0 μm, *R*_on,sp_ showed increasing trends from 1.94 mΩ·cm^2^ to 1.96 mΩ·cm^2^. The effect on the *R*_on,sp_ brought by the variety of *L*_channel_ was little. The growth of *N*_a_ could enlarge the *V*_BR_ from 65 to 2632 V under 0.1 um. *V*_BR_ increased and saturated at *L*_channel_ = 0.8 μm under *N*_a_ = 2.0 × 10^17^ cm^−3^. In contrast, *V*_BR_ increased and saturated at *L*_channel_ = 0.2 μm under *N*_a_ = 8.0 × 10^17^ cm^−3^ and *N*_a_ = 1.0 × 10^18^ cm^−3^, respectively. Moreover, *V*_BR_ kept saturated from 0.1 μm to 1.0 μm of *L*_channel_ under *N*_a_ = 3.0 × 10^18^ cm^−3^. The high enough value of *L*_channel_ · *N*_a_ could achieve avalanche breakdown, which could be seen from the saturated *V*_BR_. On the contrary, the unsaturated *V*_BR_ was known as punch-through breakdown. Similarly, the variation trend of FOM was the same as *V*_BR_. The peak FOM obtained was 3.59 GW/cm^2^. *V*_th_ grew from 1.19 V to 7.93 V under *L*_channel_ = 1.0 μm with the increase of *N*_a_ as shown in Fig. 8b. The change of *N*_a_ had a marked effect on *V*_th_, whereas the impact brought by the variety of *L*_channel_ on *V*_th_ was negligible.

The impact of the *L*_dielectric_ was researched based on *L*_channel_ = 1.0 μm. The growth of *L*_dielectric_ would reduce *C*_ox_ and electron concentration of channel layer under on-state condition, resulting in larger *R*_on,sp_ and *V*_th_. *R*_on,sp_ increased from 1.92 mΩ·cm^2^ to 2.24 mΩ·cm^2^ and *V*_th_ enhanced from 1.87 V to 8.97 V for *L*_dielectric_ increasing from 8 to 50 nm as shown in Fig. 8c, d. *L*_dielectric_ had no effect on *V*_BR_, leading to the reduction of FOM from 3.63 GW/cm^2^ to 3.10 GW/cm^2^.

## Conclusion

In this work, we analysed the performance of the fabricated GaN TG-MOSFET on 4-inch free-standing GaN substrate by Silvaco TCAD. The mechanisms of on-state and breakdown have been well studied. The key device parameters have been thoroughly researched considering the trade-off between *R*_on,sp_ and *V*_BR_. The normally off operation and high breakdown voltage show enormous potential to provide a bright future application for vertical GaN-based high power electronics.

## Data Availability

All data generated or analyzed during this study are included within the article.
